# Commentary: Two approaches to analyze platform trials incorporating non-concurrent controls with a common assumption

**DOI:** 10.1177/17407745221112016

**Published:** 2022-08-22

**Authors:** Marta Bofill Roig, Franz König, Elias Meyer, Martin Posch

**Affiliations:** Section for Medical Statistics, Center for Medical Statistics, Informatics, and Intelligent Systems, Medical University of Vienna, Vienna, Austria

Marschner and Schou^1^ and Saville et al.^2^ propose methods to incorporate non-concurrent controls in the analysis of platform trials. Both approaches are designed to adjust for potential time trends and are based on the same underlying principle: the data from all treatment arms are used to estimate potential time trends, and these estimates are used as a correction to adjust the estimates from non-concurrent controls. Saville et al.’s time machine uses a Bayesian time trend estimate for the adjustment. Marschner and Schou’s network-meta analysis approach, that is based on treatment effect differences compared to other treatment arms, implicitly adjusts the control response with a time trend estimate obtained from a linear model.

Besides the Bayesian time machine, Saville et al. also consider what they call a time categorical model, a linear model that models the outcome with the independent factors treatment (a categorical variable) and time (a categorical variable, where time, is divided into intervals). The time categorical model resembles the network meta-analytic approach of Marschner and Schou which is also based on linear model estimates. A difference in the approaches is the definition of the time intervals. While Saville et al. use fixed time intervals of equal length, the time intervals in the models of Marschner and Schou are defined by the time points where treatments enter or leave the platform. A similar time categorical model with periods defined by the entry and exit times of treatments in the platform was already considered by Lee and Wason^3^ and further investigated by Bofill Roig et al.^4^

As in the Bayesian time machine, the time categorical model and the network analysis approach are based on the same principle to adjust for time trends; they rely on similar assumptions. For the time machine, it is assumed that “any temporal drift applies to entire population (and all treatment arms)” and that “the treatment effect for each intervention is constant across time on the linear predictor scale.” For the network approach, a similar assumption is stated, namely, “that the underlying difference between two treatments is identical for all direct and indirect comparisons between the two treatments.” Note that for logistic regression models, the latter means that the differences are equal on the logistic predictor scale modeling the log odds. Thus, both approaches rely on the assumption of equal time trends on the model scale. For a trial without interim analyses or other adaptations, Bofill Roig et al.^4^ showed that under this assumption (and the standard assumptions for linear or logistic regression models), the time categorical model (where time intervals are defined by the exit and entry of treatment arms) gives valid treatment effect estimates. Under equal time trends models with time categorized in fixed time intervals in general will also lead to (asymptotically) unbiased estimates, if the length of the intervals converges to zero (which, however, will result in a larger variance). In addition, the Bayesian time trend estimate is valid only if the smoothing parameter for the time trend estimate is chosen correctly. However, if the condition of equal time trends across arms is violated, the treatment effects may be biased and the type 1 error rate substantially inflated, regardless how the time intervals are defined.

Marschner and Schou refer to the scenario of different time trends as “inconsistency” defined as “an interaction between treatment comparisons and the stage-specific design.” Thus, an inconsistency occurs if the difference between treatment effects differs between time periods which correspond to different time trends across treatments. How plausible such an interaction depends on the specific scenario.

Platform trials can be susceptible to time trends, for instance due to their long run time, for example, if populations vary over time or there are changes in the recruitment centers (see Sridhara et al.^5^). As discussed above, the models can appropriately adjust for time trends that affect all arms equally. However, if the time trends differ (on the model scale) between arms and there are interactions between treatment effect and time, the time trend adjustment of the considered approaches may fail. Furthermore, interim analyses and other adaptations may, in addition, lead to bias and type 1 error rate inflation if not accounted for.

Thus, for the application of the approaches, it is essential to rule out potential interactions between treatment effects and time. How plausible such interactions will depend on the specific setting. In a dynamic setting such as the SARS-CoV-2 pandemic, differences in time trends can be caused, for example, by a change of the disease due to different variants of the virus or shifts in population which are affected.

Saville et al. as well as Marschner and Schou note that the condition of equal time trends can be empirically tested. The latter also gives an example from the STAMPEDE trial. There, the estimated treatment effects based on concurrent and non-concurrent controls differ substantially, and the null hypothesis of equal treatment effects is even rejected in a statistical test.

It is noted, however, that trials are usually not powered to detect differences in treatment effects over time (or equally different time trends across treatments). Therefore, unless differences in time trends are very large, they may remain undetected and lead to biased estimates and inflated type 1 error rates.

When time trends are caused by changes in the patient population which are reflected by observed baseline covariates, one can directly adjust for these. This can be implemented by extension of both of the considered approaches. However, such adjustments rely on model assumptions.

In conclusion, unbiasedness and type 1 error rate control of analyses including non-concurrent controls ultimately rely on assumptions regarding the time trends. These assumptions need not only be assessed by examination of the trial data but may need primarily to be justified by subject matter knowledge. If this is not possible, an analysis using concurrent controls can be the more reliable choice.
